# Incidence of calcaneal apophysitis (Sever’s disease) and return-to-play in adolescent athletes of a German youth soccer academy: a retrospective study of 10 years

**DOI:** 10.1186/s13018-022-02979-9

**Published:** 2022-02-09

**Authors:** Patrick Belikan, Lars-Christopher Färber, Frédéric Abel, Tobias E. Nowak, Philipp Drees, Stefan G. Mattyasovszky

**Affiliations:** Department of Orthopaedics and Traumatology, University Medical Centre of the Johannes Gutenberg-University of Mainz, Langenbeckstraße 1, 55131 Mainz, Germany

**Keywords:** Sever’s disease, Calcaneal apophysitis, Heel, Heel pain, Athletic injury, Youth, Return to play, Soccer

## Abstract

**Background:**

Calcaneal apophysitis, or Sever's disease, is the most common cause of heel pain in childhood and adolescence. It is regarded as an overuse syndrome. Studies on the incidence of calcaneal apophysitis in young athletes and their associated return-to-play time are lacking in the current literature. The aim of our current study was to identify the incidence of calcaneal apophysitis in professional youth soccer, the associated time to return-to-play, predisposing factors and their impact on time to return-to-play.

**Methods:**

Retrospective evaluation of injury data gathered from a German youth soccer academy in the years 2009–2018. In total, 4326 injury cases in 612 players were included in the study. The diagnosis and the follow-up visits were carried out in a weekly consultation hour at the youth academy.

**Results:**

During the observation period of 10 years, 22 cases of calcaneal apophysitis were detected. The incidence of calcaneal apophysitis per 100 athletes per year was found to be 0.36. The mean age of the affected athletes at the time of diagnosis was 11.8 ± 2.1 years (MW ± SD). The complaints were unilateral in 20 and bilateral in two cases. Three of the 22 detected cases of calcaneal apophysitis (13.6%) were recurrent injuries. The mean time to return-to-play of the affected athletes was 60.7 ± 64.9 days (MW ± SD). Athletes with recurrent complaints showed longer recovery time and time to return-to-play when compared to players with primary diagnosed disease. Our results could show that neither age nor body mass index at the time of diagnosis had an impact on time to return-to-play.

**Conclusions:**

This is the first study investigating the incidence of calcaneal apophysitis and the associated time to return-to-play in youth elite soccer. Calcaneal apophysitis results in substantial time loss for the athletes. Further prospective clinical studies are required to fully understand the etiology and risk factors for calcaneal apophysitis and therefore develop preventive strategies.

## Introduction

Calcaneal apophysitis—also known as Sever’s disease—is the most common cause of posterior heel pain in children and adolescents [1]. Calcaneal apophysitis is regarded as an overuse syndrome caused by repetitive microtrauma resulting from an increased traction impact on the calcaneal apophysis by the calf muscles through the Achilles tendon. This in order leads to calcaneal apophysis damage with small avulsion fractures followed by local inflammation [2–9]. According to the current literature, overpronation of the foot, flatfoot, tightness of the plantar fascia or the Achilles tendon, running on hard surfaces and inappropriate footwear are additional risk factors promoting disease development [10].

Calcaneal apophysitis occurs during the years of skeletal development where the calcaneal apophysis is open [11]. The calcaneal apophysis appears at around 7–9 years of age and fuses between the ages 15–17 [2, 9, 12–14]. Typically, children and adolescents between the ages of 8 and 15 years are affected by the disease [2, 10, 15]. After closure of the calcaneal apophysis, full recovery of calcaneal apophysitis is expected [1, 16].

Patients typically present with tenderness and pain on palpation at the bony insertion site of the Achilles tendon at the calcaneus. The affected heel often proves to be clinically inapparent without significant swelling. Tightness of calf muscles and the Achilles tendon as well as weakness on dorsiflexion are further characteristic clinical findings [9]. The onset of calcaneal apophysitis is primarily insidious. The symptoms are aggravated by physical activity and sports [2, 3, 6, 17–19] and are often occurring during growth spurts and at the beginning of the sporting season [6]. In advanced cases the pain may lead to the avoidance of heel load during gait on the involved limb, a limp or even pain at rest [2, 17]. Calcaneal apophysitis has been shown to have a substantial impact on health-related quality of life [20, 21].

For diagnosis, a structured history and physical examination are sufficient [2, 6, 18, 22]. An important clinical test is the ‘squeeze test’ (manual medial and lateral compression of the heel) [23]. In some cases, depending on the history of the patient, magnetic resonance imaging (MRI) is recommended to rule out other potential differential diagnoses, such as tumors or infections [5].

The main goal of the initial therapy of calcaneal apophysitis is pain relief. For most cases, rest alleviates pain. Therefore, limiting strenuous activity, especially jumping and running, is paramount. In the primary phase cooling and non-steroidal anti-inflammatory drugs (NSAID) therapy are recommended, followed by a stretching program that focuses on the calf muscles and helps to enhance dorsiflexion of the ankle joint [1, 10]. It has been reported that orthotics like heel lifts, heel cups, and heel pads reduce axial loads and traction forces on the apophysis of the heel, thus leading to decreased symptoms [1, 9]. In cases of severe symptoms with persistent pain and limping gait, a short period of cast immobilization or a walking boot can be helpful [9, 24, 25]. There are only limited reports of complications when following this treatment regimen [26]. Usually, patients are pain-free within a period of a few weeks to several months. Return-to-play (RTP) ranges from two to eight weeks [10, 15].

Our study sought to identify the incidence of calcaneal apophysitis, the return-to-play (RTP) of athletes diagnosed with calcaneal apophysitis in competitive youth soccer and to determine risk factors for longer RTP.

## Materials and methods

The underlying study is a retrospective case series. Neither ethics approval nor consent to participate were required.

Medical data of 612 male soccer players of a professional German soccer youth academy in the period of 2009–2018 were investigated and manually scanned for calcaneal apophysitis. In total 4326 injury cases in players aged 7–19 were included in the study.

In all included cases diagnosis was made by experienced team physicians after a detailed history and physical examination, including the aforementioned squeeze test. Foot biomechanics were assessed in the standing player, and abnormalities as flatfoot and hyperpronation were documented. For all players body mass index (BMI) was calculated as body weight normalized by height squared (${\text{BMI}} = \frac{{{\text{body }}\;{\text{weight}}\; \left( {{\text{kg}}} \right)}}{{{\text{height}}\;\left( {\text{m}} \right)^{2} }})$. Calculated BMI is the basis of WHO’s definition of overweight (25 ≤ BMI < 30) and obesity (BMI ≥ 30).

Follow-up visits were performed every 1–3 weeks. The first day of symptoms and the day of RTP were precisely documented, and the loss of time was calculated. Individual data of all included athletes were transferred in a table sheet. The affected side, the patients’ age and the BMI at the time of diagnosis were also documented. Data sheets with incomplete documentation (*n* = 3) were excluded.

The treatment strategy in all cases was rest, ice, seven-day course of oral non-steroidal anti-inflammatory drugs, manual therapy with detonization of the calf muscles and a standardized stretching program, followed by step-by-step return to activity. Foot orthotics were prescribed when athletes showed biomechanical abnormalities of the foot. Athletes were cleared for competition as soon as they were free of pain and their function was normal compared to contralateral side.

According to the injury registration consensus [27] injury severity was categorized in minimal (1–3 days); mild (4–7 days); moderate (8–28 days); severe (> 28 days).

The incidence of calcaneal apophysitis per 100 athletes per year was calculated as follows:

$${\text{Incidence}}\;{\text{ per }}\;{100 }\;{\text{athletes }}\;{\text{per }}\;{\text{year}} = \left( {\frac{{{\text{all}}\;{\text{ cases }}\;{\text{of }}\;{\text{calcaneal }}\;{\text{apophysitis}}\;{\text{ from}}\; 2009\;{\text{to}}\;2018}}{{{\text{all athletes from}}\; 2009\;{\text{to}}\;2018}}} \right) \times \frac{{100 \;{\text{athletes}} }}{{10\;{\text{years}}}}.$$

Statistical analysis was carried out using GraphPad Prism 9 (GraphPad Software). Results are shown as mean ± standard deviation, unless indicated otherwise. Normal distribution of continuous variables (BMI, age, RTP) was evaluated using normal probability plots and the D’Agostino-Pearson-Test with variable age normally and variables BMI and RTP not normally distributed. For normally distributed variables an unpaired t-test and for not normally distributed variables a Mann–Whitney-*U*-test was performed. Linear regression and Pearson r test were performed to evaluate the association between age, RTP and BMI. A *p* value of < 0.05 was considered significant.

## Results

22 cases of calcaneal apophysitis were diagnosed in 19 athletes. Thus, 0.51% of all musculoskeletal complaints were diagnosed as calcaneal apophysitis. The players’ demographics are shown in Table 1.

**Table 1:** Demographics of athletes diagnosed with calcaneal apophysitis

Variable	
Number of calcaneal apophysitis diagnosis	*n* = 22
Number of affected athletes	*n* = 19
Age, years	11.77 ± 2.11
Minimum	8
Maximum	17
Biomechanical abnormality of the foot (flatfoot, hyperpronation)	*n* = 6 (31.6%)
BMI (kg/m^2^)	17.25 ± 1.89
Age and BMI are presented as mean ± SD	

**Fig. 1:**
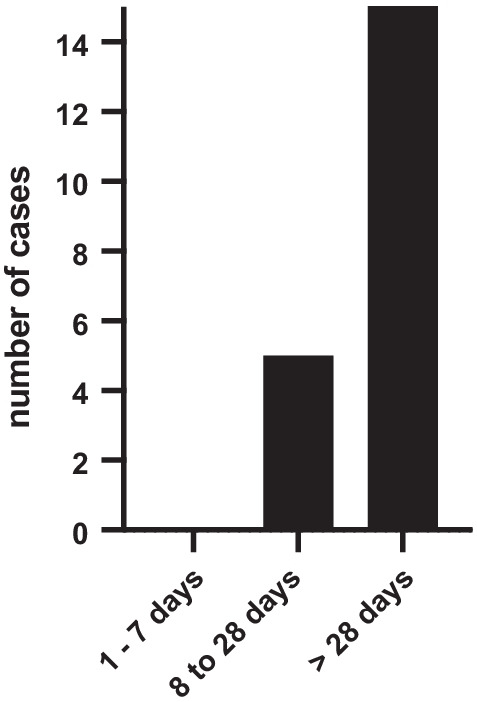
Distribution of return-to-play in athletes diagnosed with calcaneal apophysitis according to the injury registration consensus [27]

The incidence of calcaneal apophysitis per 100 athletes per year in our cohort was found to be 0.36. Two out of 19 athletes (10.5%) suffered from bilateral apophysitis calcanei, whereas the rest was affected unilaterally (Table 2). Five patients (26.3%) showed flatfoot and hyperpronation deformities, whereas 14 patients (73.7%) had normal biomechanics of the foot. Recurrent diagnosis could be observed in 3 athletes (15.8%). One of the athletes was affected on the left foot twice. Another athlete was initially affected on both sides and at the recurrent diagnosis showed the symptoms only on one side. The third athlete was affected on one side at initial diagnosis and on both sides at the recurrent diagnosis. The mean RTP in all cases of calcaneal apophysitis was 60.7 (CI 32.0–89.5) days (see Table 2). No injury was classified as minimal or mild, 5 (22.7%) as moderate and for 17 cases of calcaneal apophysitis (77.3%) RTP was longer than 4 weeks (severe injury) (see Fig. 1).

**Table 2:** Mean age and return-to-play in Athletes diagnosed with calcaneal apophysitis

	*n* (%)	Age (years)	RTP (days)
Calcaneal apophysitis cases	22 (100)	11.8 (10.8–12.7)	60.7 (32–89.5)
Primary diagnosis	19 (86)	11.7 (10.6–12.8)	41.7 (31.1–52.3)*
Recurrent diagnosis	3 (14)	12.3 (8.5–16.1)	181.0 (− 120.2 to 482.2)
Single limb affected	20 (91)	11.9 (10.8–12.9)	45.9 (31–60.7)*
Both limbs affected	2 (9)	11 (− 1.7 to 23.7)	209.5 (− 1080 to 1499)
Biomechanical normal foot	16 (73)	12.2 (10.9–13.4)	64.9 (24.6–105.3)
Biomechanical abnormality of the foot	6 (27)	10.7 (10.1–11.2)	49.5 (33.7–65.3)

95% Confidence intervals are shown in parentheses if not indicated otherwise

RTP was longer in patients suffering from bilateral apophysitis compared to unilaterally affected athletes (209.5 vs. 45.9 days; *p* = 0.017) and patients with recurrent calcaneal apophysitis showed longer RTP when compared to first diagnosed calcaneal apophysitis (181.0 vs. 41.7 days; *p* = 0.002) (see Table 2).

In our study, we did not find a correlation between the time to RTP and the age at the time of diagnosis (*p* = 0.97). Furthermore, there was no correlation between the BMI and the time to RTP (*p* = 0.54) (data not shown).

**Fig. 2:**
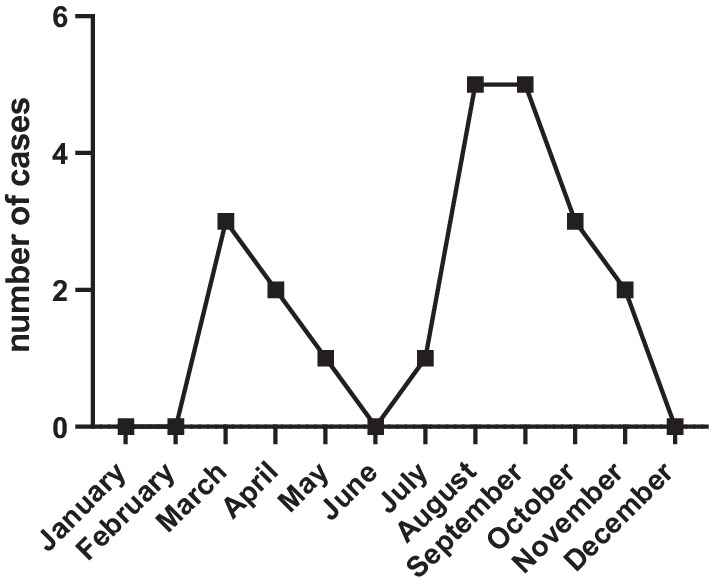
Monthly distribution of calcaneal apophysitis diagnosis

Interestingly, we could show that most cases of apophysitis calcanei occurred in the early stages of the season or after winter break (see Fig. 2).

## Discussion

Calcaneal apophysitis is the most common cause of heel pain in childhood and adolescence and accounts for 8% of all pediatric overuse injuries [9, 24]. In professional youth soccer not only the medical staff, but also the young athletes, their parents and representatives of the clubs take a great interest in the incidence and expected downtime regarding certain disorders. So far there is a lack in the literature pertaining to this exact information. This study aimed to fill this gap in evidence by investigating the incidence and loss of time until RTP of calcaneal apophysitis in young professional soccer players.

Athletic activity has been identified as a major risk factor [15, 21]. Athletes participating in sports requiring jumping, running and plantar-flexion activation, including soccer, gymnastics, dance, track and basketball, are at the highest risk to develop calcaneal apophysitis [7, 9, 12, 25, 28]. In a retrospective study by McKenzie, all cases of calcaneal apophysitis were involved in running-related sports [29]. Micheli et al. found 29% of calcaneal apophysitis patients were involved in soccer, gymnastics, running and basketball [2]. Children taking part in competitive sports are more likely to develop calcaneal apophysitis [15]. We therefore addressed the question of incidence of calcaneal apophysitis in athletes of a professional soccer youth academy.

In our cohort study, the incidence of the disease per 100 athletes per year was found to be 0.36. In athletic children and adolescents, it is expected to be higher as in the general population [2, 6, 17–19]. We could show an incidence of calcaneal apophysitis of 5.1 per 1000 musculoskeletal complaints in academy soccer, whereas incidences of around 3.5 in 1000 patients are reported for the general population [5, 18]. Incidence of calcaneal apophysitis between 2 and 22% among all musculoskeletal injuries were reported [6, 7, 17, 29–32]. Our study’s ratio of calcaneal apophysitis in musculoskeletal complaint was 0.51% and therefore less than in other reported studies.

De Inocencio et al*.* reported that 5% of musculoskeletal complaints in a pediatric clinic could be attributed to calcaneal apophysitis [31]. Ovara described the 6-year incidence to be 22.7% [32]. The higher incidence in the aforementioned studies may be due to the fact that these pediatric clinics usually see patients preselected by general practitioners. Data of specialized clinics are neither appropriate for depicting the general incidence in a population nor to be compared to our data as all athletes introduce themselves—without exception—to the team physician during the weekly consultation hour.

Another reason for the lower incidence found in our study is that athletes in the soccer academy receive better and more intensive care with physiotherapy and prevention training compared to children at the same age participating in hobby sports. Easier access to physiotherapy (i.e., stretching, detonization), the aforementioned intensive care as well as highly educated coaches can positively influence disease development in professional soccer academies.

The age of onset of calcaneal apophysitis in our cohort was between eight and 17 years. This is in accordance with the current literature regarding the appearance of the calcaneal apophysis and the onset of calcaneal apophysitis in other studies [2, 9, 10, 12–15].

Ceylan and Caypinar reported a possible association of Sever’s disease with physical activity by detecting more diagnosis during the spring season [5]. We can support this thesis with our present study as a majority of the cases could be seen within the first three months of the season or after winter break.

Bilateral presentation of calcaneal apophysitis is described in more than half of the cases [5, 33]. In our study bilateral occurrence was only observed in 10.5% of all athletes. Even with bilateral occurrence, one side becomes symptomatic earlier than the other. The lower rate of bilateral occurrence in our collective might be due to the earlier identification of the overuse syndrome by well-equipped specially trained medical staff. When unilateral symptoms occur, an athlete may be rested even before the other affected side becomes symptomatic.

High BMI is considered a risk factor of calcaneal apophysitis [21, 34]. We therefore hypothesized that an increased BMI leads to a longer period to RTP. In our study none of the athletes with calcaneal apophysitis was classified as overweight based on the international BMI cut-off points (BMI > 25 kg/m^2^). Moreover, there was no correlation between the BMI and RTP in athletes with calcaneal apophysitis.

Overpronation of the foot and flat-foot are considered as risk factors for developing calcaneal apophysitis [10, 33]. 26.3% of patients in our study presented biomechanical abnormalities of the foot. The overall prevalence of biomechanical abnormalities in childhood and adolescence is reported to be 10 – 70% in general population [35]. We did not find any correlation between the biomechanical abnormalities of the foot and RTP in our Sever’s patients.

Pain associated with calcaneal apophysitis can last from a few weeks to several months [15]. Return to sports is reported to be mostly within the range of two to eight weeks [10]. Our Sever’s patients returned to play after a mean total of 8.7 weeks. The minimum time loss in our study was 14 days and a maximum of 311 days was recorded in a case with bilateral complaints.

We saw a tendency that bilaterally affected players were longer absent from the pitch compared to unilaterally affected athletes. Furthermore, athletes with recurrent calcaneal apophysitis showed longer RTP. One might expect younger patients to show prolonged complaints of calcaneal apophysitis due to their longer periods of growth [15]. However, we could not observe a correlation between the RTP and the age at time of diagnosis.

Our study has some major limitations that have to be considered. A general limitation is the retrospective design with high risk of data loss. Although not common in the academy, athletes with the disease may have seen their general practitioner primarily and were therefore not documented in our database. Furthermore, not all athletes with complaints may have presented at the club’s consultation hours, as only half of the patients with musculoskeletal complaints do consult a physician [36]. For that reasons, the real incidence may be higher than that detected in our study.

A further limitation is patient’s compliance during therapy. Athletes sometimes turn to several doctors, so that therapy becomes intransparent and uncontrolled. Not to mention, failure to rest from activities at school and in the players free time may also lead to prolonged periods of pain. Lack of compliance and communication with the athlete can result in greater loss of time. As follow-up visits were performed every one to three weeks, we cannot rule out that a high percentage of players have rested longer than necessary.

Missing imaging and a diagnosis based on history and clinical examination may also be considered a limitation. Thus, in the absence of typical findings during physical examination but with a fitting patient history as well as rapid improvement in the course of the disease, a nonspecific heel pain may have been diagnosed in which, however, the exact differential diagnosis of apophysitis could have been made by appropriate imaging. This in turn could lead to a lower incidence.

Similarly, changes within the physiotherapy staff may have resulted in a bias regarding the incidence of apophysitis, as changes in preventive measures could result in an increase or decrease of cases.

## Conclusion

To our knowledge, this is the first study quantifying the incidence of calcaneal apophysitis in competitive youth soccer players. According to our statistics, the incidence of calcaneal apophysitis in youth soccer players is not significantly higher when compared to the general population. As expected, the incidence in our study group was also lower than in specialized pediatric clinics due to selection bias. Recurrent or bilateral diagnosis seem to be risk factors for longer RTP. The underlying study is the first to evaluate RTP in young academy soccer players with calcaneal apophysitis. This vital information provided to the athletes, their parents and the coaches alike can help deal with the unpleasant condition that is calcaneal apophysitis.

## Data Availability

The datasets used in this study are available from the corresponding author on reasonable request.

## Abbreviations

BMI: Body Mass Index
NSAID: Non-steroidal anti-inflammatory drugs
RTP: Return-to-Play
